# Neurologists' Assessment of Mental Comorbidity in Patients With Vertigo and Dizziness in Routine Clinical Care—Comparison With a Structured Clinical Interview

**DOI:** 10.3389/fneur.2018.00957

**Published:** 2018-11-13

**Authors:** Karina Limburg, Andreas Dinkel, Gabriele Schmid-Mühlbauer, Heribert Sattel, Katharina Radziej, Sandra Becker-Bense, Peter Henningsen, Marianne Dieterich, Claas Lahmann

**Affiliations:** ^1^LMU Munich, Department of Psychology, Munich, Germany; ^2^Department of Psychosomatic Medicine and Psychotherapy, Klinikum Rechts der Isar, Technical University Munich, Munich, Germany; ^3^Landeshauptstadt München, Munich, Germany; ^4^German Center for Vertigo and Balance Disorders, University Hospital, Ludwig-Maximilians-University, Munich, Germany; ^5^Department of Neurology, University Hospital, Ludwig-Maximilians University, Munich, Germany; ^6^Cluster of Systems Neurology—SyNergy, Munich, Germany; ^7^Department of Psychosomatic Medicine and Psychotherapy, University Medical Center Freiburg, Freiburg, Germany

**Keywords:** structured clinical interviews for mental disorders, vertigo, dizziness, psychiatric disorders, diagnostic agreement

## Abstract

**Background:** Mental health comorbidities are frequent in patients with vertigo and dizziness. The current study was conducted in a specialized interdisciplinary university center for vertigo and dizziness. Clinical routines consist of a structured work-up in which neuro-otological and neurological tests are performed to first detect possible organic vestibular deficits. In addition, psychiatric disorders and comorbidities are considered. The study aimed to evaluate neurologists' awareness of psychiatric next to somatic disorders within patients' first examination in terms of diagnostic congruence between neurologists' diagnoses and structured clinical assessment of mental disorders.

**Methods:** The study involved 392 patients. Diagnostic evaluation included (a) structured history-taking (including psychosocial anamnesis), neurological, and neuro-otological diagnostics conducted by neurologists and (b) a structured clinical interview for mental disorders (SCID-I) conducted by psychologists and final-year medical or psychology students. Cohen's Kappa was calculated to determine agreement rates regarding depression and anxiety disorders; additionally, sensitivity and specificity were evaluated.

**Results:** Neurologists' assessments led to at least one psychiatric diagnosis among the main diagnoses in 40 (10.2 %) patients, whereas the structured clinical interview led to at least one DSM-IV psychiatric diagnosis in 174 (44.4%) of the patients. Agreement was low (κ < 0.2); sensitivity was low (15%) but specificity was high (98%).

**Conclusions:** Agreement between the diagnosis of neurologists and structured clinical interviews for psychiatric disorders is low. Since psychiatric disorders are frequent in vertigo and dizziness and tend to take a chronic course, improving early recognition and implementing appropriate care concepts is vital.

## Introduction

Vertigo and dizziness are frequently presented symptoms that are comorbid with mental disorders in about 30–50% of complex and often chronic vertigo and dizziness syndromes (1, 2). The most common comorbidities are depression and anxiety (3). Additionally, functional vertigo and dizziness symptoms (i.e., symptoms without any underlying structural dysfunction) are very common and often occur due to a psychiatric condition (4, 5). Initially, patients with vertigo and dizziness symptoms usually consult their general practitioner or a specialist from a somatic discipline, typically a neurologist or otolaryngologist. Since mental comorbidities often tend to take a chronic course if not recognized early (6, 7), detecting possible mental disorders early in the diagnostic process would be beneficial in order to initiate the appropriate treatment or prevention methods. However, even though mental comorbidities are frequent in many neurological disorders and are thought to complicate treatment (8), few investigations toward neurologists' awareness of psychiatric conditions in clinical routine exist. One study examining this issue found low sensitivity in neurologists' detection of depression in patients with Parkinson's symptoms (9). Findings from respective studies in other medical fields, e.g., in oncology (10), primary and secondary care regarding alcohol problems (11), and other somatic hospital departments (12) agree that the recognition and correct identification of psychiatric conditions or psychological distress is low across the disciplines. Therefore, collaborative care approaches that aim at integrating psychosomatic or psychiatric assessments and care into somatic care are becoming increasingly popular since they could assist in recognition and treatment of mental health conditions (13, 14).

The aim of the current study was to investigate neurologists' recognition of psychiatric diagnoses in patients with vertigo and dizziness symptoms during clinical routine, i.e., a first examination at a specialized tertiary care center. To do so, we aimed to evaluate the prevalence rates estimated by the primary diagnoses of neurologists and compare them with those detected by the current gold standard, the structured clinical interview for mental disorders after DSM-IV, SCID-I (15). In this regard, we aimed at specifically evaluating the concordance of anxiety and depressive disorder diagnoses since these are most common (3, 16).

## Methods

### Study design, setting, and sample

This secondary cross-sectional analysis of a longitudinal study was conducted between May 2010 and June 2012 and involved 687 patients who gave their informed consent (from a total of 860 eligible patients). Patients were recruited through routine care appointments at the interdisciplinary German Center for Vertigo and Balance Disorders, University Hospital Munich, Germany. Due to organizational reasons (e.g., living outside of Munich or nausea and vomiting after caloric testing), not all patients finished the time consuming psychological assessment following the neurological and neuro-otological procedures. Therefore, we were able to include and analyse complete data from 392 patients (44.1% male, 54.8 ± 16.0 years of age). The majority of these patients, i.e., almost 80 % presented with chronic complaints that had persisted for at least 6 months before initial presentation at the center (17).

Patients were included if they were at least 18 years of age and were fluent with the German language. Furthermore, assessment via SCID-I and completed relevant questionnaires needed to be available. Exclusion criteria were any of the following diagnoses: neurodegenerative disorder (e.g., dementia), schizoaffective disorder, psychotic disorder, substance abuse, or severe suicidal tendencies. All participants were informed about the aims of the study and provided written informed consent. The study was approved by the Ethics Committee of the University of Munich (ref: 108–10). The design of this study has been previously described in detail by Lahmann et al. (18).

### Neurological diagnostic work-up

All patients underwent structured history taking and a systematic and standardized physical examination by neurologists at the German Center for Vertigo and Balance Disorders, including complete neurological, neuro-otological, neuro-ophthalmological, and neuro-orthoptic examination. Details on the neurological assessments are provided elsewhere (17). History taking included a psychosocial anamnesis to evaluate mental health comorbidity in an unstructured manner. However, neurologists were not explicitly instructed to screen for psychiatric disorders since the first neuro-otological examination aims at disclosing vestibular dysfunctions. Psychiatric diagnoses were assessed according to the International Classification of Diseases [ICD-10; (19)]. All diagnoses, i.e., main and secondary diagnoses, were noted in a specified section within the patients' reports; these were extracted for the current study. However, some physicians noted aspects of psychopathology (i.e., depressed mood, thought disorders, etc.) in the running text of the patient's report which were not coded in the diagnoses section.

Neurologists working at the center are residents in their second or higher year of specialized medical training. The residents are supervised by a senior neurologist specialized in neuro-otology with whom every diagnostic decision is discussed. At the time of data collection, there were seven residents and five senior neurologists working at the center. The neurologists are clinically trained in the screening of mental disorders via standardized clinical teachings and courses within the field of psychosomatic medicine, which are held by colleagues from the psychosomatic department.

### Psychometric examination: structured clinical interview (SCID-I)

Psychologists and final-year medical or psychology students conducted structured clinical interviews (SCID-I) to assess patients' mental disorders and psychiatric comorbidity according to the DSM-IV classification system independently of their diagnoses given by the neurologists (15). Interviews were conducted on the same day after the neurological assessment. We used the DSM-IV classification system since data collection was conducted before the DSM-5 (20) came out. Furthermore, the DSM-IV is still the current clinical “gold” standard and no German translation of the SCID for DSM-5 exists yet. However, because most DSM-IV somatoform disorders have been merged into DSM-5 somatic symptom disorder (SSD) (20), we decided not to evaluate the concordance of DSM-IV somatoform disorder diagnoses from our evaluation. All interviewers underwent intensive training, which included practice interviews with patients that were not recruited for the study. Interrater reliability was evaluated via interviews with a simulated patient; Kappa was 0.94. Interviewers were required to attend a regular SCID supervision led by a senior physician (CL).

### Statistical analysis

We conducted all analyses using SPSS (version 24). We used descriptive statistics to describe the sample and to evaluate prevalence rates of diagnoses. A sensitivity analysis comparing study sample and dropout group was conducted with *t*-tests and chi-square tests. To test for differences among neurologists' psychiatric diagnoses vs. current SCID-I diagnoses, chi-square tests were conducted. Furthermore, the agreement rate between the diagnosis given by neurologists after clinical diagnostic work-up and the SCID-I diagnosis was evaluated using Cohen's Kappa coefficient (K), which was computed as follows: [(p_o_-p_c_)/(1-p_c_)], i.e., p_o_ is the observed agreement rate and p_c_ the agreement rate by chance. Cohen's Kappa can be classified as follows: K ≤ 0.20 no or weak agreement; 0.21 ≤ K ≤ 0.40 small agreement; 0.41 ≤ K ≤ 0.60 moderate agreement; 0.61 ≤ K ≤ 0.80 high agreement; 0.81 ≤ K ≤ 1.00 very high agreement (21). In addition, we investigated sensitivity and specificity of doctors' assessments as indicators of the true positive and true negative rates, respectively (22).

## Results

Sociodemographic and medical characteristics of the patient study sample (*n* = 392) and the group excluded from the analyses (*n* = 295) along with a sensitivity analysis regarding relevant variables are presented in Table 1. Study sample and excluded patients differed regarding age (with the study sample being significantly younger). In the neurological diagnostic workup, a total of 252 patients (64.3%) of the study sample were diagnosed with a purely structural cause of vertigo. For the remaining 140 patients (35.7%), the vertigo and dizziness symptoms were classified by the neurologists as having functional vertigo or a functional component. About a third of patients received more than one somatic diagnosis, thus 468 diagnoses were given overall. Of those, 144 (30.8%) were functional vertigo and dizziness, 324 (69.2%) included a structural dysfunction amongst the main diagnoses.

**Table 1:** Sociodemographic and medical characteristics of the study sample and patients excluded from the analyses.

	**Study sample (*n =* 392)**	**Excluded from analyses (*n = *295*)***		***X^2^* or *T***
		***n* (existing data)**	***M* (*SD*) or *n* (*%*)**
**VARIABLE**				
Age, *M* (*SD*)	53.9 (15.8)	120	57.5 (16.9)	*T* = 2.2^*^
Female gender, *n* (%)	221 (56.4)	295	180 (61.0)	*X^2^ =* 1.5
Marital status (*n %* married)	246 (62.8)	119	74 (62.2)	*X^2^ =* 8.2
Education		120		*X^2^ =* 1.4
9th grade or less, *n* (%)	161 (41.1)		50 (41.7)
10th grade, *n* (%)	124 (31.6)		36 (30.0)
High school graduate, *n* (%)	45 (11.5)		14 (11.7)
University graduate, *n* (%)	62 (15.8)		20 (16.7)
Neurological diagnoses, *n (total)*	468	295	367
Functional vertigo and dizziness symptoms, *n* (%)	144 (30.8)		119 (32.4)	*X^2^ =* 0.9
Vestibular paroxysmia, *n* (%)	30 (6.4)		20 (5.4)	*X^2^ =* 0.19
Vestibular migraine, *n* (%)	75 (16.0)		41 (11.2)	*X^2^ =* 3.3
Multisensory deficit, *n* (%)	23 (4.9)		27 (7.4)	*X^2^ =* 2.7
Benign paroxysmal positional vertigo, *n* (%)	66 (14.1)		45 (12.3)	*X^2^ =* 0.31
Central vertigo, *n* (%)	24 (5.1)		25 (6.8)	*X^2^ =* 1.4
Meniere's disease, *n* (%)	57 (12.2)		44 (12.0)	*X^2^ =* 0.02
Vestibular neuritis, *n* (%)	22 (4.7)		12 (3.3)	*X^2^ =* 0.85
Bilateral Vestibulopathy, *n* (%)	27 (5.8)		34 (9.3)	*X^2^ =* 4.5^*^
**PSYCHOPATHOLOGY**				
Depression (BDI-II), *M* (*SD*)	11.4 (8.2)	112	11.9 (9.1)	*T* = 0.61
Anxiety (BAI), *M* (*SD*)	13.2 (9.4)	115	14.8 (10.6)	*T* = 1.53
Somatization (PHQ-15), *M* (*SD*)	9.8 (4.9)	116	9.7 (5.0)	*T* = −0.22

*Multiple psychiatric and neurologic diagnoses were allowed if indicated*.

*DSM, Diagnostic and Statistical Manual of Mental Disorders; PHQ, Patient Health Questionnaire; BDI, Beck Depression Inventory; BAI, Beck Anxiety Inventory*.

* *p < 0.05*.

### Prevalence and agreement of psychiatric diagnoses: neurologists' diagnoses compared to SCID-I

Table 2 presents the psychiatric diagnoses given by neurologists and those after DSM-IV and their prevalence rates. Structured clinical interview led to a total of 275 diagnoses and at least one psychiatric diagnosis after DSM-IV in 174 (44.4%) of all patients; 66 (16.8%) patients of the total sample fulfilled DSM-IV-criteria of a depressive disorder, 130 (33.2%) patients fulfilled the criteria of at least one anxiety disorder. The neurologists' assessment led to substantially lower rates of psychiatric diagnoses: 49 diagnoses were given in total, 40 (10.2%) of all patients were diagnosed with at least one psychiatric diagnosis, 15 (3.8%) patients of the whole sample with a depressive disorder and 24 (6.1%) with at least one anxiety disorder. Remaining diagnoses were substance-related and adjustment disorders. Agreement between the neurologists' diagnoses and structured clinical interviews was low throughout, with Cohen's κ values below 0.2. A total of 10 (15.2%) depressive and 20 (15.3%) of all anxiety disorders were correctly identified by neurologists (see Table 3). For depression, sensitivity of doctors' diagnoses was 15.2%, specificity was 98.5%. Rates were similar for anxiety disorders, with a sensitivity index of 15.4% and specificity of 98.5%.

**Table 2:** Diagnoses according to neurologists and DSM-IV.

**Diagnosis**	**Neurologist *n* (% of total)**	**DSM-IV *n* (% of total)**
Total number of psychiatric diagnoses	49 (100)	275 (100)
Depressive disorder	15 (30.6)	66 (23.9)
Anxiety disorder	27 (55.1)	169 (61.5)
Panic disorder	15 (30.6)	23 (8.4)
Panic disorder with agoraphobia	2 (4.1)	25 (9.1)
Agoraphobia without panic disorder	2 (4.1)	40 (14.6)
Social phobia	0 (0.0)	14 (5.1)
Specific phobia	1 (2.0)	48 (17.5)
OCD	0 (0.0)	0 (0.0)
PTSD	0 (0.0)	9 (3.3)
GAD	2 (4.1)	10 (3.5)
Not otherwise specified	5 (10.2)	0 (0.0)
Substance-related disorder	1 (2.0)	31 (11.3)
Eating disorder	0 (0.0)	9 (3.3)
Adjustment disorder	6 (12.3)	0 (0.0)

*OCD, obsessive-compulsive disorder; PTSD, posttraumatic stress disorder; GAD, generalized anxiety disorder*.

**Table 3:** Agreement between doctor's rating of psychiatric disorders and DSM-IV-classification of disorders (excluding somatoform disorders).

**DSM-IV**	**Doctor's rating**		***X*2**	**Cohen's κ**
	**No (*n*)**	**Yes (*n*)**		
**ANY PSYCHIATRIC DIAGNOSIS GIVEN**				
No (*n*)	210	8	22.9^***^	0.16
Yes (*n*)	142	32	
**DEPRESSIVE DISORDER**				
No (*n*)	321	5	27.7^***^	0.20
Yes (*n*)	56	10	
**ANY ANXIETY DISORDER**				
No (*n*)	258	4	29.0^***^	0.17
Yes (*n*)	110	20	

*DSM, Diagnostic and Statistical Manual of Mental Disorders*.

*** *p < 0.001*.

## Discussion

We investigated neurologists' awareness of psychiatric comorbidities in patients with vertigo and dizziness symptoms at their first examination at a tertiary care center specialized in vertigo and balance disorders. Our main finding was that the recognition of psychiatric disorders amongst neurologists in routine clinical practice is very low with only about 10% of all patients being diagnosed with a psychiatric disorder by the neurologist vs. about 44% fulfilling DSM-IV criteria. Further, only 15% of depressive and anxiety disorders were identified correctly, i.e., in accordance with DSM-IV criteria, among all patients. On one hand, corresponding specificity rates were high, indicating that when the neurologists make a psychiatric diagnosis, it mostly is correct. On the other hand, however, doctors in our investigation had a tendency not to give or not to document psychiatric diagnoses in a high number of cases as depicted by the low sensitivity rates.

Our findings of low agreement between SCID-I and doctors' ratings relate to previous studies in neurology (8, 9) and other medical fields (10–12) as well as to meta-analytic findings (23). Agreement rates also correspond to findings comparing standardized and unstandardized methods concerning patients with psychiatric disorders (24–28). Standardized interviews such as the SCID-I are seen as the “gold standard” to diagnose mental disorders (29). However, they are highly time as well as cost consuming and thus may not be conducted as a routine diagnostic interview in primary, secondary, or tertiary care. This was also true for the primary routine diagnostics in the current study.

It is important to note that the study was conducted in routine clinical practice, i.e., neurologists were trained to screen for relevant mental disorders, but not explicitly instructed to regularly perform a related questionnaire. Thus, it may be that neurologists were aware of mental psychopathology and simply did not note their impression in the file, especially when a structural dysfunction was found. Moreover, there were structural barriers for the documentation of psychiatric disorders in routine clinical practice. Aspects of psychopathology may have been described in the doctor's report but were not captured by the current study since only actual diagnoses were evaluated. Hence, although our results likely underestimate the diagnostic competency of neurologists during their clinical practice in a tertiary care center, an adequate testing for psychiatric comorbidities and disorders is mandatory and must be included in the diagnostic workflow. In this regard, one has to differentiate between secondary and tertiary care. In secondary care, one would likely assume that a neurologist is practicing within the standards of neurological care even if psychiatric diagnoses are missed in individual patients. In tertiary care, however, the finding that mental health comorbidities that affect 30–50% of patients are very often not detected is concerning. As it is not realistic to perform detailed standardized interviews for mental disorders by a psychiatrist or psychologist in a routine setting, one solution to this issue could be the implementation of a screening procedure by application of a validated, standardized short questionnaire (e.g., Beck Depression Inventory [BDI; (30)], Beck Anxiety Inventory [BAI; (31)], Hospital Anxiety and Depression Scale [HADS; (32)] , Somatic Symptom Disorder-B Criteria Scale [SSD-12; (33)]. This could help to improve the awareness of mental disorders and the rate of early diagnosis and treatment. However, it goes without saying that self-report screening questionnaires do not lead to greater awareness of and better care for mental health comorbidities without equally implementing changes in clinical workflow in order to ensure that mental health symptoms are adequately dealt with. In this regard, research in cardiovascular care has shown that screening for depression does not improve outcome for heart failure patients (34, 35). Further, a review on recommendations for depression screening in primary care found that patients who undergo screening do not have better outcomes (36) and authors of a meta-analytic study on screening tools for depression in non-mental health settings recommend only using these tools if appropriate organizational strategies and modifications are available (37). Similar findings have been made in oncology, where evidence suggests that a positive screening for psychological distress often does not lead to appropriate care, although a referral is mandatory for the screening to be effective in improving patients' psychological outcome (38). A strategy to better deal with mental health comorbidities in somatic care in general and neurological care specifically could be collaborative care approaches. Clinical guidelines for managing functional symptoms already recommend a collaborative stepped care approach in which the primary care physician coordinates treatment procedures (39). Collaborative care concepts have been implemented and evaluated for depression and somatoform disorders, results indicate that they are superior to treatment as usual (13, 40). For patients with somatoform and functional disorders, the evaluation of a pre- and post-intervention in primary care indicated that the investigated interdisciplinary network (The Network for Somatoform and Functional Disorders, Sofu-Net) was feasible and helped to improve doctor-patient communication about psychosocial distress (14). These collaborative care approaches have in common that they have their starting point in primary care. Patients at our center are usually referred from primary to secondary care, the latter then refers to our tertiary treatment. In contrast, an example of a stepped collaborative care concept for our patient group analogous to the Sofu-Net (14) could look as follows. The patient would first visit the primary care physician (PCP). Based on this visit, the PCP could decide to refer to secondary care (e.g., a neurologist). After this, PCP and secondary care physician would discuss the findings and could then decide to (a) observe the symptoms over a defined period of time or (b) refer the patient to our center. Patients could be screened for mental health comorbidities at any of these stages, but at the latest at our center. If the screening is positive, the PCP should be informed and initiate appropriate treatment, i.e., next to somatic care, psychotherapeutic treatment should be initiated. This would require a care network that enables the PCP to refer patients to psychotherapy and be informed about the progress of therapy. This concept could apply to patients presenting with both chronic and acute symptoms and would ensure that patients with chronic complaints are always screened for mental comorbidity. Those with acute symptoms would likely more rarely present at the tertiary center. If they do present, they would also be screened for psychopathology which would help to recognize the symptoms at an early stage and therefore simplify access to treatment if necessary, even if the vertigo and dizziness symptoms resolve after a relatively short period of time.

The advantage of the current study is that we examined a large sample size and used multiple sources of information and diagnostic assessment methods such as routine clinical work-ups and standardized interviews for mental disorders. However, there are some limitations: Our sample is representative for a specialized tertiary care center but not for all patients with vertigo and dizziness. Thus, a selection bias must be assumed with respect to chronification and a higher rate of psychiatric disorders.

In conclusion, our results show that neurologists' awareness of psychiatric disorders in routine clinical practice in a specialized tertiary care center for vertigo and dizziness is low. This is in accordance with previous studies in both similar as well as different medical fields. However, particularly since psychiatric disorders tend to take a chronic course if not recognized early (6, 7), diagnosis of psychopathology should be given more importance and appropriate concepts of care, such as stepped collaborative care, should be implemented.

## Data availability statement

The raw data supporting the conclusions of this manuscript will be made available by the authors, without undue reservation, to any qualified researcher.

## Author contributions

The study was designed by GS-M, CL, MD, PH, and SB-B. KL, GS-M, SB-B, and KR led the data collection. KL conducted the statistical analysis and wrote all drafts. AD, HS, GS-M, and KR contributed to the analysis strategy and all drafts. All authors contributed to the interpretation of the results. All authors critically reviewed the manuscript and approved the final version. All authors agree to be accountable for all aspects of the work in ensuring that questions related to the accuracy or integrity of any part of the work are appropriately investigated and resolved.

### Conflict of interest statement

The authors declare that the research was conducted in the absence of any commercial or financial relationships that could be construed as a potential conflict of interest.
